# Safety and Feasibility of Hippocampal Sparing Cranial Radiation in Pediatric and Adolescent Acute Lymphoblastic Leukemia Patients: A Prospective Study

**DOI:** 10.7759/cureus.62715

**Published:** 2024-06-19

**Authors:** Ambedkar Yadala, Ashutosh Mukherjee, Vijayaprabhu Neelakandan, Arivazhagan Karunanithi, Biswajit Dubashi, Vikas Menon, Thiraviyam Elumalai, Deepak Bharathi, Bhargav S Gundapuneedi, Vignesh Loganathan

**Affiliations:** 1 Radiation Oncology, Jawaharlal Institute of Postgraduate Medical Education and Research, Puducherry, IND; 2 Radiation Oncology, Homi Bhabha Cancer Hospital and Mahamana Pandit Madan Mohan Malaviya Cancer Center (MPMMCC) Tata Memorial Center, Varanasi, IND; 3 Clinical Psychology, Jawaharlal Institute of Postgraduate Medical Education and Research, Puducherry, IND; 4 Medical Oncology, Jawaharlal Institute of Postgraduate Medical Education and Research, Pondicherry, IND; 5 Psychiatry, Jawaharlal Institute of Postgraduate Medical Education and Research, Puducherry, IND; 6 Clinical Oncology, Cambridge University Hospitals NHS Foundation Trust, Manchester, GBR; 7 Radiology, Jawaharlal Institute of Postgraduate Medical Education and Research, Puducherry, IND; 8 Community Medicine, Jawaharlal Institute of Postgraduate Medical Education and Research, Pondicherry, IND

**Keywords:** neurocognitive tests, vmat radiotherapy, cranial irradiation, hippocampal sparing, acute leukaemia

## Abstract

Introduction

Acute lymphoblastic leukemia (ALL) constitutes a significant portion of pediatric malignancies, with central nervous system (CNS) relapse posing a considerable threat to patient outcomes. While cranial radiation therapy (CRT) has been utilized to mitigate CNS relapse, it is associated with neurocognitive (NC) side effects. This study explores the feasibility and safety of using volumetric arc therapy (VMAT) with hippocampal sparing (HS) during cranial radiation therapy for ALL patients, aiming to reduce these side effects.

Methodology

This prospective observational study included pediatric and young adult patients with ALL who were in remission. HS was achieved using VMAT, and NC assessments were performed at baseline, six months, one year, and, to a limited extent, four years posttreatment.

Results

VMAT enabled precise hippocampal-sparing CRT with minimal dose to the hippocampus. Dosimetric analysis revealed that patients receiving 18 Gy had mean doses to planning target volume (PTV) and bilateral hippocampus of 18.9 and 9 Gy, respectively. Those receiving 12 Gy had corresponding doses of 13.3 and 7 Gy, respectively. Conformity and homogeneity indices were 0.9 and 0.1, and no brain relapses were observed among the patients in this study. NC assessments demonstrated no decline in intelligence quotient (IQ) scores over time, while only a subset of patients could be assessed at the four-year mark; telephone interviews suggested no significant cognitive decline.

Conclusions

This study highlights the potential of VMAT with HS as a promising approach to CRT for ALL patients in reducing the risk of NC side effects. The absence of brain relapses and preservation of NC function are encouraging findings, though larger studies are necessary to establish conclusive evidence.

## Introduction

Leukemia is one of the most common pediatric malignancies worldwide, with approximately 0.5 million new cases annually [1]. In India, the overall proportion of leukemia among childhood cancers ranges from 26.7% to 52.3% [2]. Historically, high relapse rates have decreased due to central nervous system (CNS)-directed treatments, including chemotherapy and cranial radiation therapy (CRT) [3]. Initial protocols used craniospinal irradiation as a CNS-directed treatment, but this led to high rates of neurocognitive (NC) impairment and secondary CNS malignancies. Chemotherapy alone results in NC late effects in 40%-60% of acute lymphoblastic leukemia (ALL) survivors [4]. Strong evidence suggests that radiation-induced damage to the hippocampus is responsible for the decline in NC function in patients who received CRT. Methotrexate has been shown to cause long-lasting suppression of hippocampal cell proliferation and impaired cognitive performance in rat models [5]. Decreased hippocampal neurogenesis is one reason for chemotherapy-induced cognitive impairment [6]. Although many protocols have excluded CRT from CNS management, it still plays a role in patients with CNS 3 [7] and those with high-risk features [8] due to the high rate of CNS relapses in these subsets. In this study, we investigated the safety and feasibility of employing hippocampal sparing (HS) techniques during CRT. We assessed NC functions before radiation therapy (RT) and post-RT at six months, one year, and four years. The earlier version of the article was previously uploaded to the Research Square preprint server on January 16, 2024, and is not currently under consideration for publication elsewhere [9].

## Materials and methods

This was a prospective observational study conducted in a regional cancer center in India, from 2015 to 2021. This study was approved by the Institute Ethics Committee (Human Studies), Jawaharlal Institute of Postgraduate Medical Education and Research (JIPMER), with proposal no. PGRMC/RT/04/2014. The patient population included pediatric and adolescent patients with ALL in remission, age range of 6-25 years. The exclusion criteria mainly included children already diagnosed with psychiatric disorders or mental retardation, and children with hypothyroidism. A total of 23 patients who fulfilled the criteria after obtaining informed consent were included in the study. All cases were diagnosed using standard diagnostic procedures and stratified according to risk criteria. These patients received a standard chemotherapy regimen according to their risk and age.

Hippocampus delineation and planning

The delineation of the hippocampi was done using the contouring guidelines outlined in RTOG 0933 on T1-weighted 3D-FSPGR (fast spin gradient) MRI axial sequences featuring a 1-mm slice thickness. Subsequently, these contours were fused with the treatment planning CT, which had a slice thickness of 3 mm.

**Figure 1:**
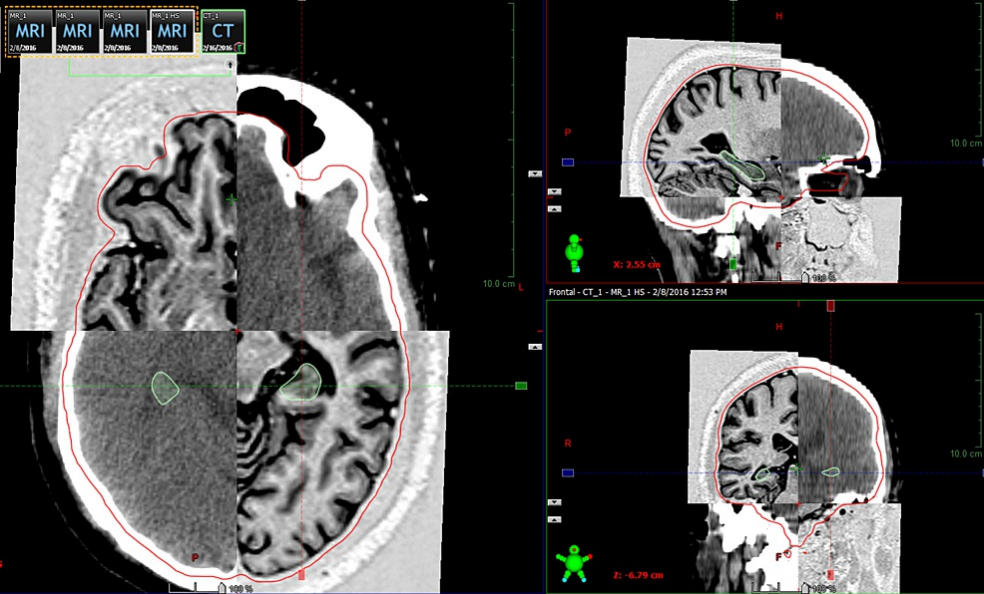
Hippocampus contouring using magnetic resonance imaging (MRI) registration. Three-dimensional image showing contouring of the hippocampus in green color.

We delineated the regions of the dentate gyrus and cornu ammonis within the hippocampal region, where neural progenitor cells crucial for memory and other cognitive functions are believed to reside (Figure 1).

**Figure 2:**
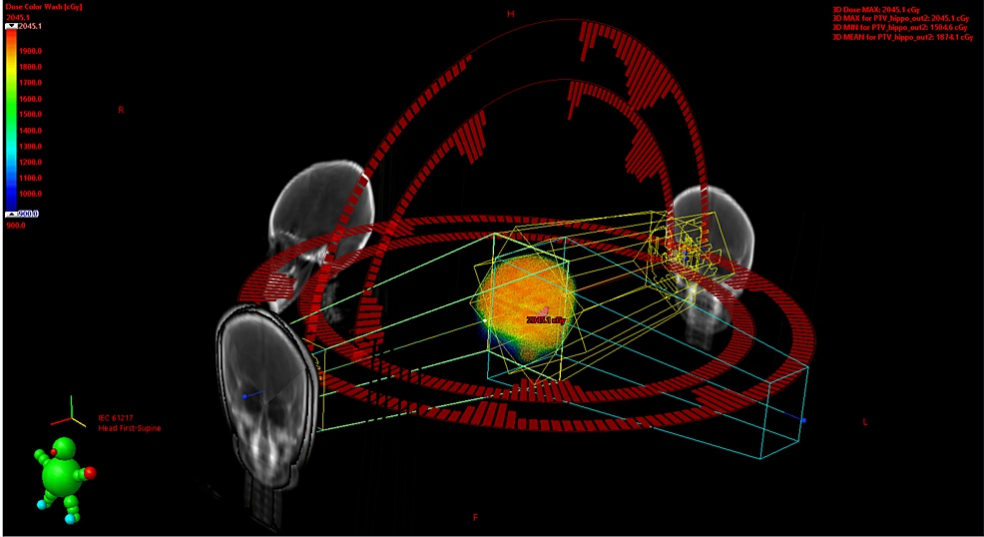
Radiation treatment planning. Planning with four arcs with two coplanar full arcs and two noncoplanar semi-arcs.

A 3 mm margin to the hippocampus was provided for planning purposes as the planning organ at risk volume (PORV). The planning target volume (PTV) was the whole brain, excluding the bilateral hippocampus. The volume of the contoured hippocampus was measured (Figure 2).

**Figure 3:**
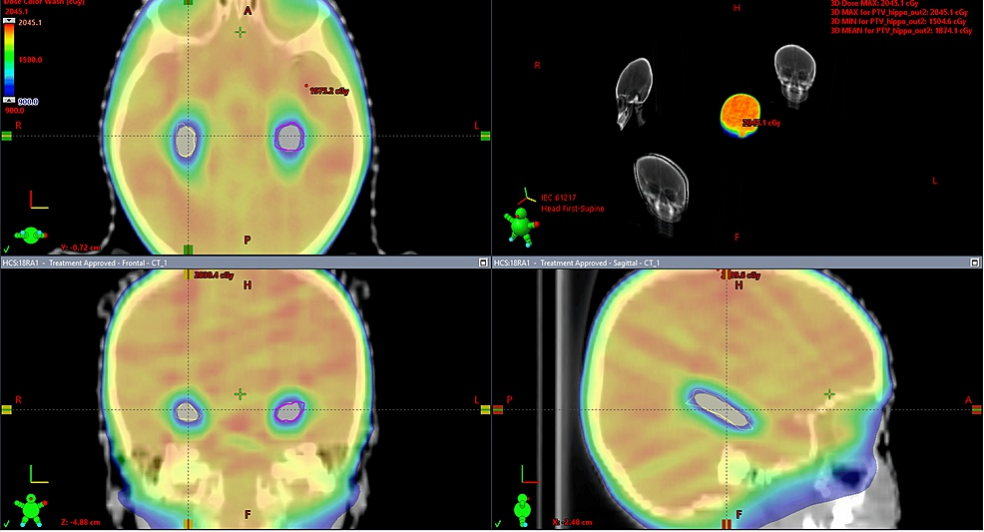
Three-dimensional treatment planning images showing dose color wash with reduced doses to bilateral hippocampi.

Planning was performed using four arcs with two coplanar full arcs and two non-coplanar semi-arcs with optimized collimator angles. All plans were completed using Eclipse Planning System version 10 (Varian Medical Systems, Inc., Palo Alto, CA) (Figure 3).

The treatment setup was performed using cone beam computed tomography (CBCT) with the on-board imager, ensuring precision with a tolerance level of 3 mm variation on the initial day of therapy. The subsequent fractions were verified using CBCT and kV images. The patient was monitored during the treatment period for any acute toxicities.

NC assessment

All patients eligible for the study were taken up for baseline NC assessments before prophylactic cranial irradiation (PCI). Patients were classified into age groups 6-15 and 16-25 years based on their age for the ease of NC assessment. NC assessments for patients aged 6-15 years were conducted using the Malin Intelligence Scale for Indian Children (MISIC) (Appendix A). This includes two sets of tests: a verbal set and performance tests. All tests were scored individually, and the sum of all the tests gave a raw score of the verbal and performance test, which was later converted to intelligence quotient (IQ) scores. The composite scores of all verbal and performance tests provided a total full-scale IQ (FSIQ) of the patient. NC assessment of patients aged 16-25 years was performed using the Wechsler Adult Performance Intelligence Scale-PR (WAPIS-PR) (Appendix B). This includes different sets of tests, including immediate and delayed recall tests, respectively. Each was scored individually. The composite score of all tests yielded a total FSIQ of the patients. All tests were administered by a trained clinical neuropsychologist. Each patient assessment took an average of three to four hours. NC assessment was performed at baseline, six months, at the end of one year, and four years. Patients who were unwilling to attend the four-year assessment due to travel and personal reasons underwent telephonic interviews to gather information regarding their academic performance and workplace efficiency.

Statistical analysis

The distribution of patients’ categorical data, such as sex and clinical characteristics, was expressed as frequencies and percentages. Data on age, CRT dosimetric parameters, and level of cognitive functions were expressed as mean with standard deviation (SD), where the sample followed a normal distribution or median with a range if it was non-normally distributed. Dosimetric parameters such as mean dose to PTV (*D*_mean_), dose received by 2% of the volume (*D*_2%_), and the dose received by 98% of the volume (*D*_98%_) were measured. The mean of all the patients with SD was recorded. Hippocampal parameters included the mean dose to the bilateral hippocampus, *D*_2%_ (dose received by 2% of the hippocampus), and *D*_98%_ (dose received by 98% of the hippocampus). The mean of all the patients was used to determine the average dose to the hippocampus. Minimal, maximal, *D*_20%_ (dose received by 20% of the hippocampus), *D*_40%_ (dose received by 40% of the hippocampus), *D*_50%_ (dose received by 50% of the hippocampus), and *D*_80%_ (dose received by 80% of the hippocampus) and doses to the combined hippocampus were expressed in mean with SD. The mean and maximum doses of the other organs at risk were measured. Survival was estimated using Kaplan-Meier survival curves. Median survival time was calculated and reported. Survival analysis was performed to estimate the incidence density of the outcome, that is, deaths along with a 95% confidence interval (CI) among the study participants. NC function differences were measured between baseline, six months, one year after, and four years post-RT. Parameters in the NC assessments were evaluated using scores to observe changes in cognitive function levels between baseline, six months, and one year after RT. Repeated measures analysis of variance (ANOVA) was performed to determine the significance of the change observed for the various parameters. A significance level of 0.05 was considered statistically significant. All analyses were performed using Stata version 14.0 (StataCorp., College Station, TX).

## Results

Patient characteristics

All 23 patients in the study underwent induction chemotherapy and received intrathecal methotrexate for CNS prophylaxis. Among these, 18 (78.3%) were treated according to the MCP-841 protocol, with a dose fractionation of 18 Gy administered in 10 fractions at 1.8 Gy per fraction. The remaining 5 (21.7%) followed the BFM-95 protocol, where the dose fractionation was 12.6 Gy in 7 fractions at 1.8 Gy per fraction. In terms of age distribution, 15 (65.2%) patients were aged 15 or older, while 8 (34.8%) were under the age of 15. Among the participants, 17 (73.9%) were male and 5 (21.7%) were female. Regarding the subtype of ALL, 5 (21.7%) had Pre-B ALL, 6 (26.1%) had T ALL, and 12 (52.2%) had B ALL. The majority of the patients (18, 78.3%) were classified as high-risk individuals.

Dosimetric data

For ease of convenience in dosimetric analysis, the study population was classified into patients who received total doses of 18 Gy and 12.6 Gy.

For patients treated with 18 Gy, the dosimetric parameters were as follows: the mean dose to PTV was 18.9 Gy ± 0.3 Gy, *D*_98%_ PTV was 17.6 ± 0.3 Gy, and *D*_2%_ PTV was 19.7 ± 0.5 Gy. The dose to the left lens of the OARs was 10.3 ± 0.8 Gy and to the right lens was 10.2 ± 0.8 Gy. The dose to OAR in the bilateral hippocampus, which is the region of interest for this study, was 9 ± 0.5 Gy. The dose to the left hippocampus was 8.9 Gy ± 0.4 Gy, and to the right hippocampus was 9 ± 0.3 Gy. The *D*_2%_ of the hippocampus was 12.6 ± 0.6 Gy. The mean of minimal and maximal doses, as well as *D*_20%_, *D*_40%_, *D*_50%_, and *D*_80%_ doses to the combined hippocampus, are also reported for 18 Gy dose fractionation. Specifically, for 18 Gy dose fractionation, the minimal and maximal doses were 6.2 Gy and 15.2 Gy, respectively. The *D*_20%_, *D*_40%_, *D*_50%_, and *D*_80%_ doses were 10.1, 9.1, 8.9, and 7.6 Gy, respectively. The conformity index (CI) and homogeneity index (HI) were 0.9 and 0.1, respectively (Table 1). 

**Table 1:** Doses received by various volumes in patients receiving 18 Gy for CRT. DMEAN PTV, mean dose to planning target volume; DMEDIAN PTV, median dose to planning target volume; D98PER PTV, dose received by 98% of planning target volume in percentage; D98 PTV, dose received by 98% of planning target volume; D95 PTV, dose received by 95% of planning target volume; D2 PTV, dose received by 2% of planning target volume; D1 PTV, dose received by 1% of planning target volume; LT HIPPO, dose received by left hippocampus; RT HIPPO, dose received by right hippocampus; HIPPOCOMBO, dose received by combined hippocampus; D2 HIPPO, dose received by 2% of hippocampus; D1 HIPPO, dose received by 1% of hippocampus; CI, conformity index; HI, homogeneity index; DHIPPO MIN, minimum dose to hippocampus; DHIPPO MAX, maximum dose to hippocampus, DHIPPO 20, dose received by 20% of hippocampus; DHIPPO 40, dose received by 40% of hippocampus; DHIPPO 80, dose received by 80% of hippocampus; CRT, cranial radiation therapy

For prescribed dose of 18 Gy (*n *= 18)	Mean	Standard deviation
LT_LENS DOSE	1030.65	88.86
RT_LENS DOSE	1021.39	82.64
DMEANPTV	1894.62	34.69
DMEDIAN PTV	2855.04	4051.62
D98 PER PTV	97.94	2.27
D98 PTV	1768.72	31.07
D95 PTV	195.92	407.99
D2 PTV	1976.44	52.89
D1 PTV	1986.02	52.66
LT HIPPO	890.20	45.24
RT HIPPO	908.51	39.45
HIPPO COMBO	903.76	58.82
D2 HIPPO	1260.35	63.13
D1 HIPPO	1867.23	2389.93
CI	0.90	0.07
HI	0.10	0.03
D HIPPO MIN	6.25	0.69
D HIPPO MAX	15.18	1.28
DHIPPO 20	10.15	0.32
DHIPPO 40	9.12	0.28
DHIPPO 80	7.63	0.58

For patients receiving 12 Gy, the mean dose to the PTV was 13.3 ± 0.2 Gy. *D*_98%_ PTV was 12.1 ± 0.3 Gy, and *D*_2%_ PTV was 13.8 ± 0.3 Gy. The dose to the OARs was as follows: left lens, 7.3 ± 0.4 Gy; right lens, 7.4 ± 0.3 Gy; bilateral hippocampus (the region of interest for this study), 7 ± 0.8 Gy; left hippocampus, 6.9 ± 1 Gy; right hippocampus, 7.1 ± 0.6 Gy. *D*_2%_ of the hippocampus was 9.2 ± 1 Gy. For 12 Gy, the minimal and maximal doses were 4.8 and 10.3 Gy, respectively. *D*_20%_, *D*_40%_, *D*_50%_, and *D*_80%_ were 7.9, 7.2, 7, and 6 Gy, respectively. The CI and HI were 0.9 and 0.1, respectively (Table 2).

**Table 2:** Doses received by various volumes in patients receiving 12 Gy. DMEAN PTV, mean dose to planning target volume; DMEDIAN PTV, median dose to planning target volume; D98PER PTV, dose received by 98% of planning target volume in percentage; D98 PTV, dose received by 98% of planning target volume; D95 PTV, dose received by 95% of planning target volume; D2 PTV, dose received by 2% of planning target volume; D1 PTV, dose received by 1% of planning target volume; LT HIPPO, dose received by the left hippocampus; RT HIPPO, dose received by the right hippocampus; HIPPOCOMBO, dose received by combined hippocampus; D2 HIPPO, dose received by 2% of hippocampus; D1 HIPPO, dose received by 1% of hippocampus, CI, conformity index;  HI, homogeneity index; DHIPPO MIN, minimum dose to hippocampus; DHIPPO MAX, maximum dose to hippocampus; DHIPPO 20, dose received by 20% of hippocampus; DHIPPO 40, dose received by 40% of hippocampus; DHIPPO 80, dose received by 80% of hippocampus; CRT, cranial radiation therapy

For prescribed dose of 12 Gy (*n* = 5)	Mean	Standard deviation
LT_LENS	735.64	43.90
RT_LENS	749.22	30.69
DMEANPTV	1330.68	22.11
DMEDIANPTV	1333.86	21.46
D98PERPTV	99.38	1.62
D98PTV	1219.06	36.66
D95PTV	101.40	1.57
D2PTV	1380.74	29.46
D1PTV	1385.70	31.02
LTHIPPO	699.56	116.73
RTHIPPO	714.02	64.97
HIPPOCOMBO	706.88	80.79
D2HIPPO	928.74	110.94
D1HIPPO	949.72	112.99
CI	0.91	0.02
HI	0.12	0.03
DHIPPOMIN	4.88	0.75
DHIPPOMAX	10.34	1.35
DHIPPO20	7.98	0.87
DHIPPO40	7.28	0.73
DHIPPO80	6.06	0.90

The mean volume of the bilateral contoured hippocampus was 4.5 cc (3.11-5.8). The average treatment time in our study was 5.5 minutes, which is less than the treatment time when delivered through intensity-modulated radiotherapy (IMRT) in a study by Nevelsky et al. [10].

Survival outcomes

**Figure 4:**
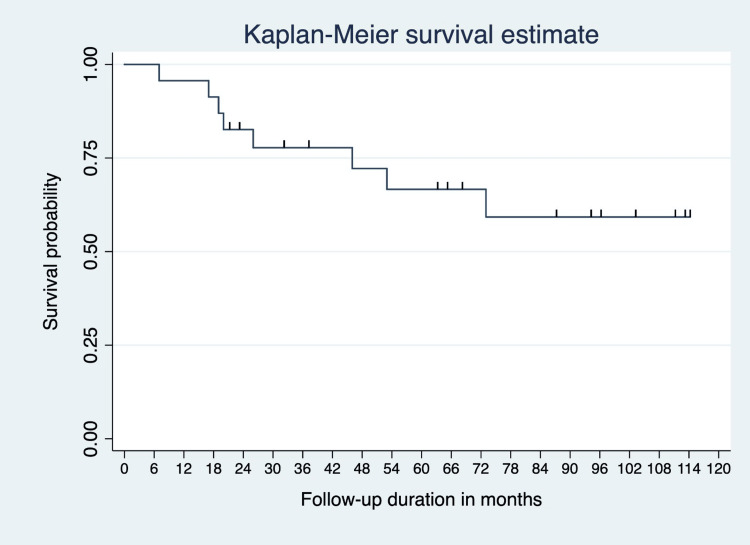
Kaplan-Meier survival estimate.

Among the 23 persons who were followed up for a total of 1,291 person-months, there were eight deaths and five lost to follow-up. The incidence rate of deaths among the sample of persons diagnosed with ALL was 5.7 deaths per 1,000 person-months (95% CI 2.9-11.5) of follow-up from the date of diagnosis. In 46 months from the time of diagnosis, about 75% of the sample of patients were alive. The median duration of survival for this sample of patients was 63 months. No CNS relapses were reported, and no patient died of CNS relapse (Figure 4).

NC analysis

Baseline NC assessment pre-RT was completed for 22 out of 23 patients. Post-RT evaluations at six months and one year were conducted for 17 patients to observe any early decline in NC functions. A four-year assessment was performed for only three patients, as the majority received local follow-up care and showed limited enthusiasm for in-person NC assessments. Instead, telephone interviews were conducted with these patients to inquire about their academic and occupational activities. For NC assessment, study participants were classified into two groups based on age and NC test: one group aged 6-15 years and another group aged 16-25 years.

For patients aged <15 years, NC assessment was performed using the Malin Intelligence Scale for Indian Children (MISIC). The mean verbal scores at baseline, six months post-RT, one year post-RT, and four years post-RT were 93, 98, 99, and 93.5, respectively, and the difference was statistically significant (*P* = 0.001). The mean performance scores at baseline, six months post-RT, one year post-RT, and four years post-RT were 92, 98, 102, and 99, respectively, and the difference was statistically significant (*P* = 0.001). The mean Auditory Verbal Learning Test (AVLT) delayed recall scores at baseline, six months post-RT, one year post-RT, and four years post-RT were 61, 76, 91, and 96, respectively, and the difference was statistically significant (*P* = 0.001). The composite score of all verbal and performance tests yielded a total FSIQ of 93 at baseline, 98 at six months post-treatment, 101 at one year post-treatment, and 95 at four years post-treatment, with a statistically significant difference (*P* = 0.001) (Table 3).

**Table 3:** Neurocognitive assessment results. ^*^Significant *P*-value.

Scores	Mean (standard deviation)				*P*-value for within patients change
Adults group (*n *= 6)					
	Baseline (*n *= 6)	At six-month follow-up (*n *= 6)	At one-year follow-up (*n *= 6)	At four-year follow-up (*n *= 1)	
Immediate recall (IR)	47.5 (±33.6)	56.7 (±27.9)	60.0 (±26.6)	95.0 (±0)	0.01*
Delayed recall (DR)	55.8 (±37.1)	67.5 (±23.4)	70.0 (±21.0)	95.0 (±0)	0.23
Intelligent quotient (IQ)	92.0 (±10.2)	99.5 (±9.3)	102.5 (±9.3)	109.0 (±0)	0.03*
Pediatrics group (*n *= 11)					
	Baseline (*n *= 11)	At six-month follow-up (*n *= 11)	At one-year follow-up (*n *= 11)	At four-year follow-up (*n *= 2)	
Verbal quotient (VQ)	93.5 (±8.8)	98.7 (±9.5)	99.9 (±9.2)	93.5 (±0.7)	<0.001*
Performance quotient (PQ)	92.6 (±10.9)	98.4 (±9.6)	102.1 (±7.8)	99.0 (±0)	<0.001*
Delayed recall (DR)	61.8 (±20.4)	76.8 (±19.1)	91.8 (±6.0)	96.5 (±0.7)	<0.001*
Full scale (IQ)	93.4 (±8.6)	98.4 (±8.0)	101.8 (±7.9)	95.0 (±0)	<0.001*

NC assessment of patients aged 16-25 years was performed using the WAPIS-PR. The mean immediate recall scores at baseline, six months post-RT, one year post-RT, and four-year post-RT were 47, 56, 60, and 95, respectively, and the difference was statistically significant (*P* = 0.01). The mean delayed recall scores at baseline, six months post-RT, one year post-RT, and four years post-RT were 55, 67, 70, and 95, respectively, and the differences were not statistically significant (*P* = 0.23). The composite score of all tests gave a total FSIQ of 92 at baseline, 99 at six months posttreatment, 102 at one year posttreatment, and 109 at four years posttreatment. The difference was statistically significant (*P* = 0.03) (Table 3).

## Discussion

CNS-directed treatment to prevent CNS relapse includes intrathecal chemotherapy and CRT. Concerns of NC sequel limit the use of CRT in the recent past. Recent studies though suggested omitting CRT, especially in low-risk and CNS 1 and 2 patients. A meta-analysis by Vora et al. showed CRT decreases relapse in CNS 3 patients [7], and some still consider it in high-risk patients [8,11]. In resource-constrained developing nations where targeted therapies, immunotherapies, and extensive ICU support for intensive chemotherapies are often unavailable or financially inaccessible, CRT remains a viable option for CNS prophylaxis. The present study aimed to evaluate the feasibility and safety of a novel approach in CRT, specifically focusing on sparing the hippocampus using the volumetric-modulated arc therapy (VMAT) technique. This innovative technique is anticipated to reduce the risk of NC side effects associated with traditional CRT.

In cases of brain metastasis and small cell lung cancer (SCLC), HS techniques using IMRT and VMAT have been tried, showing decreased doses to the hippocampus. The single-arm phase II RTOG 0933 trial demonstrated less NC decline (NCD) with HS whole brain radiotherapy (HS-WBRT) compared to a historical WBRT cohort [12]. Furthermore, data from the randomized phase III RTOG 0614 trial, although formally negative, showed that adding memantine to WBRT extended the time to NCD as a secondary endpoint [13]. Sparing the hippocampus during prophylactic cranial irradiation (PCI) better preserves cognitive function in patients with SCLC, with no observed differences in brain failure, overall survival (OS), and quality of life (QoL) compared to standard PCI [14]. Data from several other studies demonstrated limiting the dose to the hippocampus during cranial irradiation results in a lower incidence of NC side effects.

This study seeks to evaluate the feasibility and safety of a novel approach to CRT, specifically focusing on sparing the hippocampus using VMAT. This innovative technique is expected to mitigate the NC side effects associated with traditional CRT.

In our study, we achieved 100% coverage of the PTV while ensuring that the mean dose to the bilateral hippocampi remained below 9 Gy. It's noteworthy that patients undergoing a fractionation of 12.6 Gy had a mean dose of 7 Gy. The mean volume of the bilateral contoured hippocampus, 4.5 cc (range 3.11-5.8), aligns with findings in similar studies [15]. Comparisons with existing literature reveal congruence with the study by Nevelsky et al., where the hippocampus *D*_100%_ mean value was 8.37 Gy, with a maximum dose mean value of 14.35 Gy [10]. A study by Rong et al. demonstrated that rapid arc achieved a mean dose of 8.6 ± 0.3 Gy and a maximum dose of 13.6 ± 1.3 Gy in an impressively short treatment time of 2.5 minutes. This efficiency is particularly noteworthy in the context of HS [16]. Our findings, consistent with those of Pokhrel et al., indicate *D*_100%_ of 8.4 ± 0.3 Gy, a mean of 11.2 ± 0.3 Gy, and maximum doses to the hippocampus of 15.6 ± 0.4 Gy. Additionally, in this study, HI and CI mean values of 0.23 ± 0.02 and 0.96 ± 0.02, respectively [17]. In line with the study by Gondi et al., our approach maintained a hippocampal dose below 9 Gy, reinforcing the correlation between a dose close to 8 Gy and associated NCD [18]. This study adds to the expanding body of evidence supporting the safety and efficacy of VMAT as the preferred technique for HS.

The main concern addressed in this study is the possibility that reducing radiation doses to the hippocampal region could lead to a higher risk of relapse. In solid malignancies, the estimated risk of parahippocampal metastasis within 5 mm of the hippocampus is 8.6%, and none of the metastases were located within the hippocampus [19]. However, in leukemias, CNS disease or relapse is predominantly characterized by CSF positivity rather than frank metastasis. From the few patients who experienced hospital mortality in our study, we observed none of them had CNS relapse. The four-year OS in our entire cohort approximated around 75%, which is in line with findings reported in the Indian literature. A study by Swaminathan et al. reported a five-year absolute survival of 39% for patients treated for ALL [20]. Incorporating a broader perspective, the OS outcomes for all patients diagnosed with leukemia, irrespective of treatment initiation or completion, were more conservatively estimated at 33% in a cohort from a single Indian institution [21]. In adolescent and young adult (AYA) ALL, Ganesan et al. reported outcomes that revealed anticipated two-year relapse-free survival (RFS), event-free survival (EFS), and OS rates of 75%, 64%, and 75%, respectively [22]. Moreover, another study from India provided similar insights into a three-year EFS and OS of 59.4% and 61.8%, respectively [23]. Our survival rates are similar to the existing Indian literature.

In our study, early changes in NC functions were assessed by testing both before PCI, six months after PCI, one year after PCI, and four years using MISIC for patients aged <15 years. Mean verbal scores (VQ), performance scores (PQ), mean delayed recall (DR), and total FSIQ scores repeated over four years showed a significant increase in scores. In adults, NC functions were assessed using WAPIS-PR. It includes immediate recall (IR), delayed recall (DR), and a composite score IQ. The immediate recall scores were maintained but were not statistically significant. The mean delayed and composite NC scores demonstrated improvement, suggesting sustained cognitive function, and this improvement was statistically significant. Both study groups exhibited increased IQ scores, likely influenced by the evaluation process and the overall condition of the patients. Repeated administration of the same cognitive tests may have contributed to score improvements over time. Initially, during baseline assessments, patients experienced irritability due to the lengthy evaluation process conducted during induction chemotherapy. However, at six months, one-year post-RT, and four years thereafter, most patients transitioned to a more comfortable maintenance phase with monthly visits and follow-ups in the absence of disease, leading to enhanced compliance and shorter assessment durations. These factors likely contributed to the observed increase in IQ scores. We were only able to conduct a four-year assessment for three patients since the majority were receiving follow-up care locally and showed limited enthusiasm for in-person NC assessments. However, conducting telephone interviews with these patients, where we inquired about their academic and occupational activities, revealed no significant adverse impact on their cognitive function. The WAPIS-PR test administered to three patients indicated that there was no observable decline in NC function when compared to their assessment at the one-year mark. As per the findings by Rajendranath et al., 15% of children diagnosed with ALL, all of whom underwent treatment following the MCP-841 protocol with cranial radiation exposure ranging from 18 to 24 Gy, exhibited NC impairment [24]. More than 10% NCD is a significant decline, whereas our study showed no significant NCD. Although our study revealed no cognitive decline during our NC assessment, the sample size remains insufficient to definitively determine the ability of this novel approach, HS-CRT, to preserve NC function. A study with a larger sample size could potentially provide a more conclusive answer to this question.

The study presented several advantages, including the successful demonstration of the feasibility of utilizing VMAT for HS in CRT. This achievement opens avenues for additional research aimed at delivering CRT to leukemia patients without jeopardizing NC function. However, limitations include a small sample size and the absence of comprehensive NC assessments, preventing firm establishment of these results or advocating for routine clinical application. The study underscores the necessity for larger scale investigations to confirm the practical utility of this approach.

## Conclusions

Our study demonstrated the feasibility of utilizing VMAT for HS-CRT, achieving full-dose coverage to the PTV while keeping hippocampal doses below 9 Gy, a threshold associated with minimal NC impact. This adds to the existing literature supporting VMAT as a feasible technique for HS-CRT. Importantly, we observed no CNS relapses in our study population, addressing concerns regarding the safety of HS-CRT in patients with leukemia. The observed potential preservation of NC function in our study prompts further investigation into whether this can be solely attributed to the lower doses delivered to the hippocampal region or if there could also be a causal association. Future studies with a larger sample size are warranted to explore this aspect more thoroughly.

## Figures and Tables

**Figure 5:**
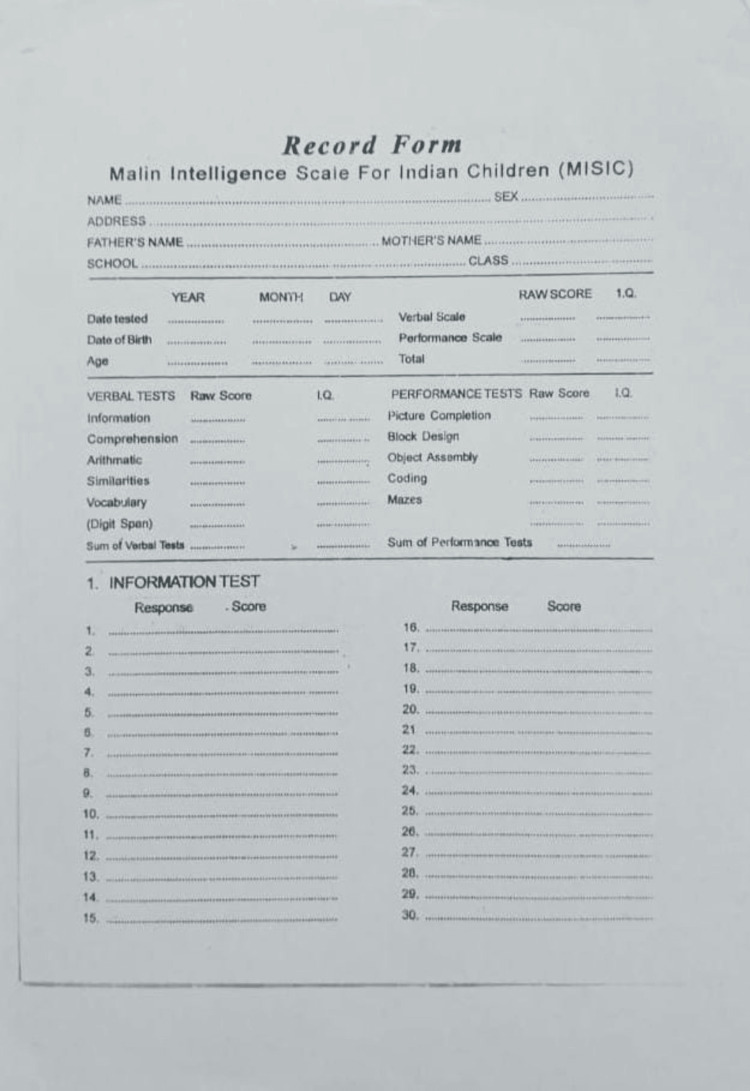
Neurocognitive assessment tool: MISIC Malin Intelligence Scale for Indian Children (MISIC) includes two sets of tests: a verbal set of tests and performance tests.

**Figure 6:**
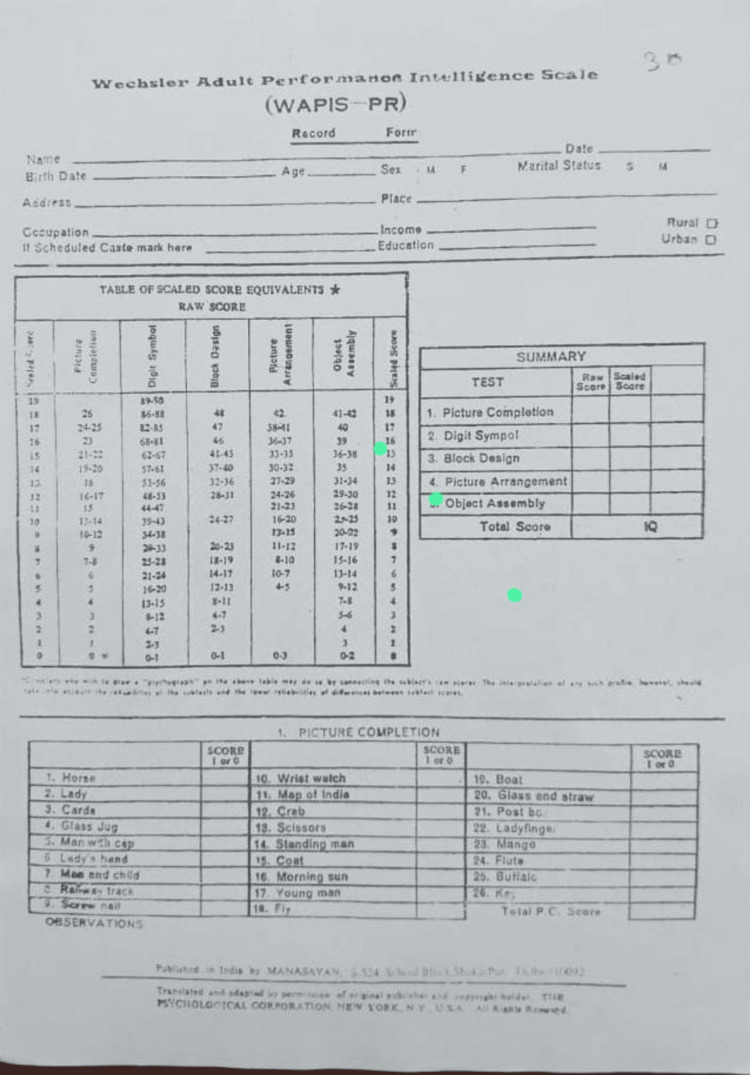
Neurocognitive tool: WAPIS-PR. The Wechsler Adult Performance Intelligence Scale-PR (WAPIS-PR) test including immediate and delayed recall tests.

## Appendices

Appendix A 

Appendix B

## References

[1] Huang J, Chan SC, Ngai CH (2022). Disease burden, risk factors, and trends of leukaemia: a global analysis. Front Oncol.

[2] Asthana S, Labani S, Mehrana S (20181). Incidence of childhood leukemia and lymphoma in India. Pediatr Hematol Oncol J.

[3] Pui CH, Cheng C, Leung W (2003). Extended follow-up of long-term survivors of childhood acute lymphoblastic leukemia. N Engl J Med.

[4] van der Plas E, Nieman BJ, Butcher DT, Hitzler JK, Weksberg R, Ito S, Schachar R (2015). Neurocognitive late effects of chemotherapy in survivors of acute lymphoblastic leukemia: focus on methotrexate. J Can Acad Child Adolesc Psychiatry.

[5] Seigers R, Schagen SB, Beerling W (2008). Long-lasting suppression of hippocampal cell proliferation and impaired cognitive performance by methotrexate in the rat. Behav Brain Res.

[6] Mounier NM, Abdel-Maged AE, Wahdan SA, Gad AM, Azab SS (2020). Chemotherapy-induced cognitive impairment (CICI): an overview of etiology and pathogenesis. Life Sci.

[7] Vora A, Andreano A, Pui CH (2016). Influence of cranial radiotherapy on outcome in children with acute lymphoblastic leukemia treated with contemporary therapy. J Clin Oncol.

[8] Sison EA, Silverman LB (2014). CNS prophylaxis in pediatric acute lymphoblastic leukemia. Hematology Am Soc Hematol Educ Program.

[9] (2024). Safety and Feasibility of Hippocampal Sparing Cranial Radiation in Acute Lymphoblastic Leukaemia Patients- A Prospective Study. Dubashi B, Menon V, et al. Safety and Feasibility of Hippocampal Sparing Cranial Radiation.

[10] Nevelsky A, Ieumwananonthachai N, Kaidar-Person O, Bar-Deroma R, Nasrallah H, Ben-Yosef R, Kuten A (2013). Hippocampal-sparing whole-brain radiotherapy using Elekta equipment. J Appl Clin Med Phys.

[11] Pinnix CC, Yahalom J, Specht L, Dabaja BS (2018). Radiation in central nervous system leukemia: guidelines from the International Lymphoma Radiation Oncology Group. Int J Radiat Oncol Biol Phys.

[12] Gondi V, Pugh SL, Tome WA (2014). Preservation of memory with conformal avoidance of the hippocampal neural stem-cell compartment during whole-brain radiotherapy for brain metastases (RTOG 0933): a phase II multi-institutional trial. J Clin Oncol.

[13] Brown PD, Gondi V, Pugh S (2020). Hippocampal avoidance during whole-brain radiotherapy plus memantine for patients with brain metastases: phase III trial NRG Oncology CC001. JCO.

[14] Rodríguez de Dios N, Couñago F, Murcia-Mejía M (2021). Randomized Phase III trial of prophylactic cranial irradiation with or without hippocampal avoidance for small-cell lung cancer (PREMER): a GICOR-GOECP-SEOR study. J Clin Oncol.

[15] Goda JS, Dutta D, Krishna U (2020). Hippocampal radiotherapy dose constraints for predicting long-term neurocognitive outcomes: mature data from a prospective trial in young patients with brain tumors. Neuro Oncol.

[16] Rong Y, Evans J, Xu-Welliver M, Pickett C, Jia G, Chen Q, Zuo L (2015). Dosimetric evaluation of intensity-modulated radiotherapy, volumetric modulated arc therapy, and helical tomotherapy for hippocampal-avoidance whole brain radiotherapy. PLoS One.

[17] Pokhrel D, Sood S, Lominska C, Kumar P, Badkul R, Jiang H, Wang F (2015). Potential for reduced radiation-induced toxicity using intensity-modulated arc therapy for whole-brain radiotherapy with hippocampal sparing. J Appl Clin Med Phys.

[18] Gondi V, Hermann BP, Mehta MP, Tomé WA (2012). Hippocampal dosimetry predicts neurocognitive function impairment after fractionated stereotactic radiotherapy for benign or low-grade adult brain tumors. Int J Radiat Oncol Biol Phys.

[19] Giuseppe ZR, Silvia C, Eleonora F (2020). Hippocampal-sparing radiotherapy and neurocognitive impairment: A systematic literature review. J Cancer Res Ther.

[20] Swaminathan R, Rama R, Shanta V (2008). Childhood cancers in Chennai, India, 1990-2001: incidence and survival. Int J Cancer.

[21] Kulkarni KP, Marwaha RK, Trehan A (2009). Survival outcome in childhood ALL: experience from a tertiary care centre in North India. Pediatr Blood Cancer.

[22] Ganesan P, Jain H, Bagal B (2021). Outcomes in adolescent and young adult acute lymphoblastic leukaemia: a report from the Indian Acute Leukaemia Research Database (INwARD) of the Hematology Cancer Consortium (HCC). Br J Haematol.

[23] Rajendra A, Jain H, Bonda VN (2021). Outcomes and prognostic factors in adolescents and young adults with ALL treated with a modified BFM-90 protocol. Blood Adv.

[24] Rajendranath R, Veeraiah S, Ramesh A, Sagar TG (2014). Late effects of treatment in survivors of childhood cancer from a tertiary cancer center in South India. South Asian J Cancer.

